# Cultural and Molecular Factors Predisposed to Non-Alcoholic Fatty Liver Disease and Type 2 Diabetes Mellitus

**DOI:** 10.3390/nu17111797

**Published:** 2025-05-26

**Authors:** Hanna George, Fajar Shodiq Permata, Crystal M. D’Souza, Ernest A. Adeghate

**Affiliations:** 1 Department of Anatomy, College of Medicine & Health Sciences, United Arab Emirates University, Al Ain P.O. Box 15551, United Arab Emirates; hanna.alain1995@gmail.com (H.G.); 700045891@uaeu.ac.ae (F.S.P.); crystal.dz@uaeu.ac.ae (C.M.D.); 2 Faculty of Veterinary Medicine, Universitas Brawijaya, Malang 65151, Indonesia; 3 Zayed Centre for Health Sciences, United Arab Emirates University, Al Ain P.O. Box 15551, United Arab Emirates

**Keywords:** culture, dietary patterns, ethnicity, NAFLD, epidemiology, diabetes mellitus, genetic pre-disposition, oxidative stress, insulin resistance, autophagy

## Abstract

There is an exponential increase in the global prevalence of non-alcoholic fatty liver disease (NAFLD) in all populations. The objective of this review is to examine how different cultures and molecular entities influence the progression of NAFLD. Research databases, including PubMed, Scopus, the American Diabetes Association, the American Liver Foundation, and Diabetes UK, were used to retrieve information. Our data analysis showed that cultural norms shape the perceptions of health, illness, and mortality, thus influencing how individuals view themselves and express their experiences and may also affect decisions related to treatment and healthcare. Cultural competence, the ability to understand and navigate cultural differences, is essential for eliciting patient and practitioner perspectives and integrating this understanding into diagnostic and treatment plans. By acknowledging and respecting a patient’s cultural background, healthcare providers can foster trust, improve care quality, enhance acceptance of diagnoses, and boost treatment adherence. Although cultural factors play a crucial role in the progression of NAFLD, the disease is also shaped by genetic predispositions, molecular mechanisms, and comorbidities. Molecular pathways involved in the development and progression of NAFLD include alterations in lipid metabolism, insulin signaling, insulin resistance, oxidative stress, defective gut microbiome, and inflammation. This study concludes that a combination of cultural preferences and molecular factors has contributed to the worldwide exponential rise in the prevalence of NAFLD, which in turn has led to an increase in the prevalence of comorbidities such as cardiovascular diseases, diabetes mellitus, and metabolic syndrome.

## 1. Introduction

Non-alcoholic fatty liver disease (NAFLD) is a complicated metabolic disorder that is rapidly acknowledged as a leading cause of morbidity and mortality, with both environmental and hereditary risk factors contributing to its occurrence [1]. Histologically, the illness can express itself in a variety of ways, from simple fatty liver to the non-alcoholic steatohepatitis (NASH) syndrome, a kind of progressive hepatic destruction by encroaching adipose tissue and inflammatory cells [1,2]. NASH patients have an increased risk of liver fibrosis, which can advance quickly from mild to severe, and eventually resulting in cancerous pathology.

## 2. History of NAFLD

According to a literature search, studies by Lonardo et al. [3] showed that, in 1836, Addison published the first description of fatty liver. Following that, several groups of pathologists embarked on finding the parallels between liver histology alterations found in diabetic and morbidly obese people and those seen in alcoholics. Rokitansky, a renowned pathologist, described a hepatic fat build-up in autopsy specimens as far back as 1838, which he hypothesized to be a contributing factor to liver cirrhosis. A diabetic patient, named Pepper, was diagnosed with liver fatty infiltrations, recorded in 1884 [3]. Bartholow wrote a study in 1885 showing a connection between being overweight and a fatty liver. In 1939, it was proposed that fatty invasion of the liver in diabetics may progress to cirrhosis [3]. There was a case report of two individuals with diabetes and fatty liver who developed a bleeding of esophageal varices, one of whom died as a result of a severe hemorrhage. Peri-lobular fibrosis of the liver has been linked to both mechanical stress and tissue anoxia in these patients. In 1962, Thaler released a second study describing the condition’s clinical and pathological characteristics. Since the 1950s, several articles have established that obese and diabetic individuals have fatty liver disease [3].

When Ludwig and his colleagues first characterized NASH in 1980, they made a distinction between the inflammatory form of fatty liver disease that appeared to be histologically similar to alcohol-induced steatohepatitis and one that was seen and identified in patients who denied having abused alcohol. The majority of the patients were overweight women, many of whom also had high blood sugar levels [4,5]. In most of the hepatic specimens, there was evidence of fibrosis with Mallory bodies and localized necrosis characterized by lobular hepatitis. In addition to being overweight, children had steatohepatitis and high liver enzyme values, and they also found nonspecific symptoms in children with steatohepatitis. The term “non-alcoholic fatty liver disease” was first introduced by Schaffner and Thaler in 1986 [6]. It is worth noting that NAFLD affects millions of people globally [2].

## 3. Methods

**Figure 1:**
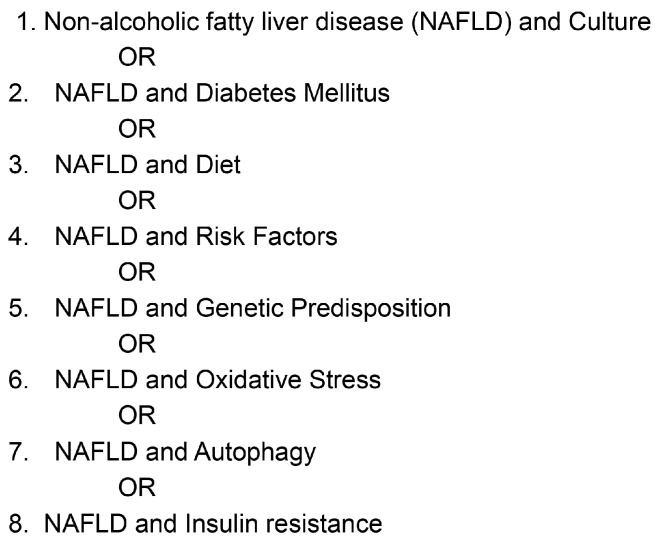
Schematic presentation of the literature search.

Research portals, such as PubMed, Scopus, the American Diabetes Association, the American Liver Foundation, Diabetes UK, and Science Direct, were utilized to source out information pertinent to this study. The keywords used in the search included: culture; diet; non-alcoholic fatty liver disease; diabetes mellitus; genetic predisposition; oxidative stress; insulin resistance; and autophagy. The strategy used for the search is given in Figure 1.

## 4. Signs and Symptoms of NAFLD

In terms of symptoms, lethargy is a prevalent complaint, although most patients are vague in terms of describing their symptoms. However, if the patient is evaluated as a whole, the symptoms of liver illness may emerge differently. However, these symptoms include but are not limited to anxiety, thirst, a shifting temperature sensation, and bloating [1,2]. Top left abdominal quadrant discomfort is an additionally prevalent complaint in these patients. The discomfort in the upper abdomen might be severe or mild. Before seeing a physician, the symptoms of recurrent bloating, discomfort, thirst, and headache were virtually always present and were frequently misdiagnosed as dyspepsia and reflux illness [1,7].

## 5. Prevalence of NAFLD

### 5.1. Global Perspectives

Since there are no universal clinical indicators for NAFLD, determining the true occurrence of the disease is difficult. However, determining the degree of fibrosis and the conclusion of the diagnosis of NAFLD requires a liver biopsy. Histopathological analysis, imaging tools, and blood tests may all be needed to make a definitive diagnosis and determine the worldwide prevalence of NAFLD [8]. According to a study and meta-analysis of the global epidemiology of NAFLD, the worldwide prevalence of NAFLD by imaging is 25.2%. However, a cheaper, more effective, and simple diagnostic tool would be needed to accurately estimate the worldwide prevalence of this common condition.

The prevalence rates of NAFLD vary from region to region. It is approximately 40% in more than half of the countries in the MENA Region [9]. This is a significant increase compared to the 25% recorded by the same group of authors in 2016 [10]. A prevalence rate of 34% was recorded for the USA [8,11,12]. In South America, a staggering rate of 59% prevalence was estimated [13]. The average prevalence of NAFLD in Asia is 29%, but with the highest rate of 51% in Indonesia [14]. The prevalence value provided for Africa ranges between 13.5% [15] and 25% [8]. The United Kingdom has a relatively low prevalence rate of 20.7% [16]. The prevalence rate recorded for Africa is probably an underestimate, as the African population consumes calorie-rich food and lives a sedentary lifestyle like their Caucasian counterparts. The frequency of NASH, on the other hand, was estimated to be lower, ranging from 3% to 5% [2].

### 5.2. Prevalence of NAFLD Amongst Different Ethnicities and Cultures in the USA

According to the most recent studies by Bonacini et al. [12], NAFLD affects around 32% of the population in the United States. While the United States is unusual in that it has a diverse mix of races and ethnicities, it also maintains a high degree of geographic and dietary uniformity, and as a result, it appears to be the best group to investigate any ethnic disparities in illness. In a population-based cohort of subjects, 23% of Hispanics had NAFLD, compared to 14% of Caucasians and 13% of African Americans, according to a new meta-analysis. When these percentages are compared to the Caucasian population, it can be determined that Hispanics have a higher relative risk of being diagnosed with NAFLD, whereas African/Americans have a relatively lower risk [12]. In the US, where NAFLD affects 20% of the people, Asians may have a lower prevalence of the condition. In a recent study on the prevalence of metabolic-associated fatty liver disease (MAFLD), a new terminology given to NAFLD in 2020 showed that the prevalence of MAFLD in the US was 34%, with the highest rate seen in Mexican Americans (54%) and the lowest in African Americans (20.5%) [16]. These values are higher than those reported by Bonacini et al. in 2021 [12] (Table 1).

## 6. Cultural Factors That Lead to the Development of NAFLD and Type 2 Diabetes in Different Ethnicities

“Culture” is defined by ideas, beliefs, attitudes, and normative or expected behavioral patterns. It permeates all that individuals do in their communities.

According to Bates and Plog [18], learning is how culture is transmitted from generation to generation.

An individual may take culture for granted and assume that their culture is always correct according to that individual. It is a complicated topic, and there is no accepted definition of it in the literature. As a result, the following definition serves as the basis for this investigation: the attitudes and beliefs that a group of people share from the same ethnic or racial background are often used to guide an individual’s conduct [19].

This section of the article focuses on various cultural factors (diet, religion, etc.) that affect the severity of NAFLD and eventually type 2 diabetes and associated syndromes.

### 6.1. Diet

#### 6.1.1. Diet and Overnutrition

Weight gain and obesity correlate well with liver steatosis. In addition, an increased intake of sugars, saturated fat, and fructose tends to increase the de novo lipogenesis in the liver, leading to the development of insulin resistance and subclinical inflammation in the liver, skeletal muscle, and adipose tissue. It is worth noting, however, that different types of diets would have different effects on the signs of NAFLD. It has recently been shown that a 12-week regimen of a Mediterranean diet significantly reduced the level of risk factors (insulin resistance, body weight, blood pressure, and blood glucose) for the progression of NAFLD [20]. In addition, animal protein is more likely to cause metabolic dysfunction-associated fatty liver disease (MAFLD) when compared to plant protein [21].

#### 6.1.2. Dietary Patterns in Asians

The most noticeable shift in diet across nations and regions within Asia is an increased intake of fats and oils, which leads to a rise in the proportion of dietary energy obtained from these components. This development has the potential to add to Asia’s rising obesity epidemic [22]. The amount of energy received from caloric sweeteners has been growing in Asian diets, and also the intake of wheat and potatoes, both of which contribute to adopting a more Western-type diet. These dietary modifications might lead to more acrylamide exposure in the diet, a proven dietary carcinogen [22]. Meat consumption is also on the rise throughout Asia, and with the International Agency for Research on Cancer naming meat and its products as some of the dietary variables linked to cancer risk, this trend is becoming a significant cause of alarm. Aside from the intake of carcinogens found in cooked and processed meat, another effect of this dietary shift is a rise in the use of processed, packaged, and convenience meals. These meals are heavy in fat, salt, and sugar. These items contribute to developing obesity and visceral fat and, eventually, fatty liver disease [22]. It is not surprising, therefore, that the prevalence of NAFLD is very high in some Asian countries, reaching a towering rate of 51% in Indonesia [14].

#### 6.1.3. Dietary Patterns in the Hispanic Population

According to the research conducted by Cuy Castellanos [23], a typical Latin American/Hispanic cuisine consists of chili, fat, cactus, coffee, rice, fowl, fish, pork, lentils, and other vegetables. Throughout Latin America, the society has developed varying dietary habits that demonstrate the nutritional differences between individuals. Hispanics with higher acculturation consume less fat and fiber than those with lower acculturation. More snacks, sweets, and deep-fried foods are also used [23], which eventually leads to the accumulation of fat in the liver.

#### 6.1.4. Dietary Patterns in Caucasian Americans

The diet of an average American is abundant in red meat, dairy goods, artificial food, and salt, alongside carbonated sweetened drinks. Based on the research conducted by Grotto and Zied [24], a total of almost 47% of the added sugars consumed come from carbonated beverages and other sweet drinks, among other things. Snacking has become a common way for Americans to consume a huge portion of their required daily calories. This trend has been on the rise in recent years [24]. Between 1978 and 2006, snacking by the adult population was forecasted to be prevalent at an increasing rate of about 97%. The total daily calories derived from the consumption of snacks increased from 18% to 24% in this period. Currently, 98% of the young population snacks, and it is estimated that snack foods account for 27% of their overall daily calorie consumption. During the last few decades, Americans have spent far more time on meal-related activities. This leads to an uninhibited and exaggerated calorie intake, resulting in obesity, NAFLD, and, eventually, type 2 diabetes mellitus [2,25].

#### 6.1.5. Dietary Patterns in African Americans

Typical African American diets are rich in unsaturated fat, sodium, and carbohydrates [26]. Those characteristics are passed down generations through recipes and preparation procedures such as soul food. In some communities, there is less food, and more people are living in poverty, which makes it more difficult for them to buy healthy alternatives to greasy and processed foods and dietary fat. It was reported that obesity affects African Americans due to limited food access. Obesity is a primary cause of NAFLD and diabetes mellitus [27].

### 6.2. Assimilation of Culture

“Acculturation” is used when a different culture adopts some aspects of another culture. For newcomers to the United States, it refers to incorporating a variety of tastes and habits from the country’s dominant culture. No standard instrument can measure acculturation. To date, no such instrument has been found. Individuals may be classified based on their self-identification, conduct, and language skills, among other factors [28].

**Figure 2:**
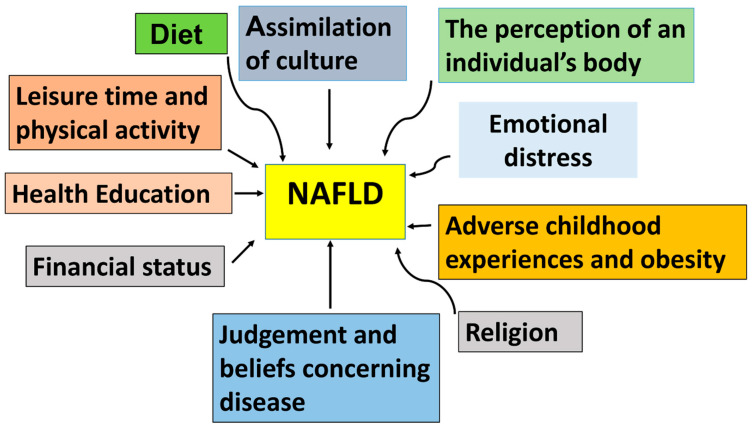
Schematic representation of cultural factors that could predispose individuals to develop non-alcoholic fatty liver disease (NAFLD).

Many studies have observed that an individual’s fondness for a specific language is a decent sign of their degree of acculturation. It is irrefutable that these variables affect clinical outcomes. Acculturation is associated with a better lifestyle and standards of living. Individuals ultimately decide what habits and preferences they want to embrace in their newfound lives. Patients should be asked about the habits they have picked up from mainstream culture, and healthcare practitioners should be receptive to this question [28]. The assimilation and acculturation of individuals into a culture where a calorie-rich diet is consumed can lead to the development of NAFLD and other metabolic diseases, such as diabetes mellitus (Figure 2).

### 6.3. The Perception of an Individual Body

Cultural differences in terms of ideal body proportions are reflected in ideal body sizes. Fallon [29] and Wiseman et al. [30] argued that, in the past, the ideal body sizes for American women have been all about being thin since the 1960s. Examples include American models, who are exemplars of idealized bodies, and models from other countries. Halliwell and Dittmar [31] found that a size 2 in women was significantly leaner than a typical American woman, having a measurement of 12–14 [31]. Some African American women in the United States may be less influenced by the ideal norm of thinness and general beauty held by the majority of white women, according to some. Caucasian Americans dominate the physical appearance of models in mass media photographs. Furthermore, in many situations of poverty, larger body shapes are valued rather than thinness. In 2010, nearly 17,000,000 women of any race lived in poverty in the USA, compared to 12.6 million males of the same race and ethnicity. The poverty rate has climbed to 25.6% in some minorities, which is higher than the national average [32]. Within these impoverished societies, bigger body types are considered representations of power, money, health, and, ultimately, a sign of physical beauty. On the other hand, being too thin is a sign of misfortune, disease, and poverty. In some instances, when someone is presented as being too thin, they are often seen as needy, suffering from hunger, the victim of substance misuse, and afflicted by illnesses such as HIV/AIDS. Therefore, the perception of an individual’s body varies among different cultures and ethnicities. All of these factors encourage individuals in these communities to gain weight, making them susceptible to developing metabolic diseases, such as NAFLD, diabetes mellitus, and hyperlipidemia (Figure 2).

### 6.4. Emotional Distress

Medical conditions and mental suffering frequently occur in tandem. Lacking adequate economic means combined with a lack of support networks from family or friends can result in feelings of loneliness, which can then evolve into depression in the long run [28]. Most of these qualities are very common among specific ethnicities, especially those who moved to the US unprepared [28].

Depressive symptoms and noncompliance with treatment plans might be exacerbated by cultural factors. Because there must be a level of adaptation to the new culture and climate, foreigners are in a more vulnerable state, where they are more susceptible to mental distress than the local population. It has been reported that depression predisposes individuals to become obese (Figure 2).

### 6.5. The Role of Adverse Childhood Experiences and Obesity

In 1985, Dr. Vincent Felitti’s San Diego-based clinic had a 50% dropout rate of patients with substantial obesity, which was a source of irritation for the doctor. Felitti was surprised to discover that the majority of dropouts were losing weight when they quit the program after conducting a review. Follow-up interviews with these patients indicated that the vast majority (55%) had been subjected to some type of sexual abuse throughout their youth. Numerous women stated that they thought their physical size assisted them in fending off unwanted sexual attempts from men [33]. Being obese gave them a chance to be protected from their destructive situation. Recently, there has been a great deal of emphasis on misuse, abandonment, and other negative childhood experiences that are linked to home dysfunction during early development, as well as poor health in adulthood, particularly extreme obesity and binge eating in adult life [33]. It turns out that most of the 286 adults who Felitti and his team interviewed had been sexually abused as children. Even though the obese individuals that Felitti interviewed were hundreds of pounds overweight, they did not consider themselves to have a health problem, as eating served as a quick solution for them [34]. Their desire at this point was to gain a level of protection from harmful predators. These observations showed that childhood experiences can negatively impact the weight of the individual, resulting in obesity, NAFLD, and diabetes mellitus (Figure 2).

### 6.6. Literacy Regarding Health Issues

Proficiency in human well-being might be portrayed as how well individuals can grasp medical problems and their outcomes.

Differences in spoken language, the inherent understanding of medical disorders, and cultural ideas about health and disease can all contribute to health consciousness. Poor health literacy is connected to age, ethnicity, socioeconomic situation, and educational level [35]. It is well known that linguistic failures have tragic consequences: poor health literacy among patients was repeatedly associated with the higher number of hospitalizations, increased use of emergency care and medication errors, as well as relatively poor health status and rates of mortality among aged people [36]. All of these could promote the severity of NAFLD and other metabolic disorders (Figure 2).

### 6.7. Judgement and Beliefs Concerning Diseases

Every social group has a set of ideas about health and sickness that they adhere to. Individuals and groups may have a distinct explanatory model of sickness that they follow [37].

Effective clinical interactions and education initiatives are dependent on health beliefs and models of how things work. Genetics, sugar consumption, stress, mental instability, and even a brief experience of fear or worry are some of the factors associated with the development of NAFLD and diabetes mellitus. Recent research investigated the various health condition attitudes and experiences of African Americans, Hispanics/Latinos, American Indians, and persons diagnosed with diabetes in a range of settings [37]. The researchers revealed the following: individuals said the current American lifestyle was to blame for their poor health, and they had lost faith in the health industry and the absence of spirituality in the current living practices [37]. This shows that the different beliefs and judgments that each patient holds regarding a disease could adversely affect the pathogenesis of NAFLD.

### 6.8. The Role of Leisure Time and Physical Activities

Lack of physical exercise is conducive to obesity, which in turn predisposes the individual to acquiring insulin resistance, NAFLD, and, eventually, diabetes [38]. Behavioral factors are also connected to an increased risk of type 2 diabetes, with aerobic and resistance exercise lowering the liver fat content by the same amount when mediated by cell dysfunction [39].

According to the National Center for Health Statistics (NCHS), adult participation in leisure-time physical exercise in the USA has declined by an average of 0.6% annually over the last 11 years. Despite their age, many people continue to engage in little to no physical activity [40].

In descending order of physical activity, it was noted that non-Hispanic white males had the highest rate of physical inactivity. It was followed by non-Hispanic black men in second position and Hispanics in third among all racial/ethnic groups.

Moreover, 33.9% of native black women, 39.6% of white women, and 21.6% of white American women were in this category [40].

Preferences for physical activity may differ between different races. Walking or dancing may be preferred by the adult Americans over aerobics and gym exercises, whereas adult Latinos preferred activities like running and going to the gym [28,41,42,43,44]. The prescription of an exercise regimen is one of the most effective ways of delaying the onset of NAFLD and diabetes mellitus.

### 6.9. Religion

Religion and faith have an impact on our daily lives. Religious traditions are manifestations of belief and deep respect for certain concepts of absolute truth that have been handed down through generations [45]. They indicate one’s position and relationship to this reality. The ultimate reality has been known by many names, including God, Atman, Nirvana, and others. It is interpreted and treated unequally by people of different religious traditions in different parts of the world [45]. In diabetes management, one prominent example of a significant effect of religion on body weight is fasting. Fasting can significantly reduce body weight and hence delay the onset of NAFLD and type 2 diabetes (Figure 2).

### 6.10. Financial Status

The level of financial difficulty an individual faces has a strong impact on the chronic diseases they have. Research shows distinctions when it comes to diabetes-related lower extremity amputation rates among adults of different races and income. This research has been demonstrated for people over the age of 50 years. The socioeconomic state is influenced by place and date of birth in the United States [46].

## 7. Molecular Mechanisms Leading to NAFLD

### 7.1. Pathogenesis of NAFLD

**Figure 3:**
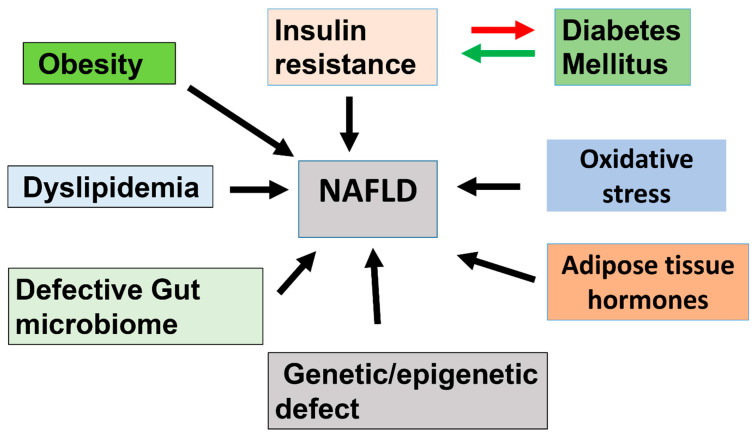
Schematic drawing of biological factors contributing to the development of non-alcoholic fatty liver disease (NAFLD).

The clear mechanism leading to the growth and development of a fatty liver is still unknown; however, a variety of biochemical pathways and the resulting liver injury represent known contributing factors. The pathogenesis of NAFLD involves inflammation and, in serious cases, fibrosis, which is usually irreversible. Hepatic triglyceride build-up causes steatosis in the liver [47]. Increased production of very low-density lipoproteins (VLDLs) and hepatic triglycerides are two possible reasons that cause hepatic steatosis (as a result of higher consumption, either a reduction in fatty acid oxidation or an increase in the supply of free fatty acids to the liver are probable explanations). Lipid peroxidation causes membrane damage, a trigger of inflammation. As a result of these alterations, hepatic stellate cells are stimulated to induce fibrosis of the liver [47]. Multiple stressors working concurrently with genetically predisposed persons to generate NAFLD are included in the “multiple hit” theory, which gives a more accurate explanation of NAFLD pathophysiology (Figure 3).

Broadly, it is sufficient to assert that NAFLD is a clinical and pathologic spectrum of liver diseases that are related to excessive fat build-up in the liver [48,49]. The spectrum of NAFLD spans through bland steatosis, steatohepatitis, and steatosis with fibrosis [50,51]. It was reported that approximately 30% of the global population and that of the United States suffer from NAFLD. However, it is largely a modifiable condition [2].

When non-alcoholic hepatic fat accumulation is connected with a condition such as the presence of substantial histological evidence of inflammatory response, such as lobular inflammation and cellular ballooning, the pathology is classified as NASH, or non-alcoholic steatohepatitis [52,53].

The chronic appearance of NASH in the presence of persistent inflammation induces fibrosis, leading to the scarring of liver tissue [52,54]. The rate at which NASH converts to cirrhosis is 7–10% per year [55,56,57,58]. The mortality rate in individuals with NASH-related cirrhosis and cirrhosis is around 2.6% and 1.4–3%, respectively [59], indicating that the mortality rate is higher in individuals with NASH. The prevalence of NAFLD can reach up to 90% in the obese population, with more than half suffering from NASH [60] (Figure 3).

### 7.2. Autophagy and NAFLD

Autophagy is an ubiquitous but essential cellular process for systematically degrading important intracellular components for recycling and energy regulation. While the majority of autophagy can be adaptive, there are also situations where self-destructive autophagic responses occur. There are three major autophagic pathways: macroautophagy, microautophagy, and chaperone-mediated autophagy [61,62]. These autophagic mechanisms have been reported in hepatocytes [63], where they help in the prevention of the development of NAFLD.

Macro-autophagy is a process whereby whole regions of cytoplasm are sequestered in double-membraned autophagosome vesicles. Autophagosomes are primarily formed by the ATG7 protein. The disruption of this pathway in Atg7^−/−^ mice leads to the accumulation of damaged organelles and altered proteins in the liver cells, be it even under normal or pathological conditions [64].

Micro-autophagy is another type of autophagy whereby the cytoplasmic organelle is engulfed directly into the lysosome by invagination of the membrane. Less selective than the amino acid motif-based pathway is chaperone-mediated autophagy, where a pattern of amino acids is identified by the chaperone rather than all motifs in general. The selection of certain proteins for transport to the lysosome is mediated by a protein known as Hsp 70 cell stress. As such, famine or inflammation may have a role in the upregulation of chaperone-mediated autophagy. The condition of getting overly stressed is termed oxidative stress [65]. It has been pointed out that autophagy is regulated by lipid metabolism. Under starvation conditions, autophagy is activated, resulting in lipolysis and the synthesis of free fatty acids, which serve as an extra source of energy for the organism. Indeed, lipophagy has been reported as a means of reducing the induction and amelioration of NAFLD [66,67,68].

This is particularly distinct because, typically, lipids are used in lipid-solvents. In the RALA255-10G hepatocyte, the availability of nutrients impairs energy production, as evidenced by the trend in lower ATP totals. Fat accumulation is mediated by Atg5, an essential gene for autophagy that has been inactivated (by a knockdown) after being treated with the fatty acid oleate, resulting in the inhibition of autophagy. Autophagy takes place when there is an availability of nutrients [69]. We have also seen that overconsumption of nutrients can be extremely deleterious because of the advent of insulin resistance, obesity, and, eventually, diabetes mellitus [2,25].

**Figure 4:**
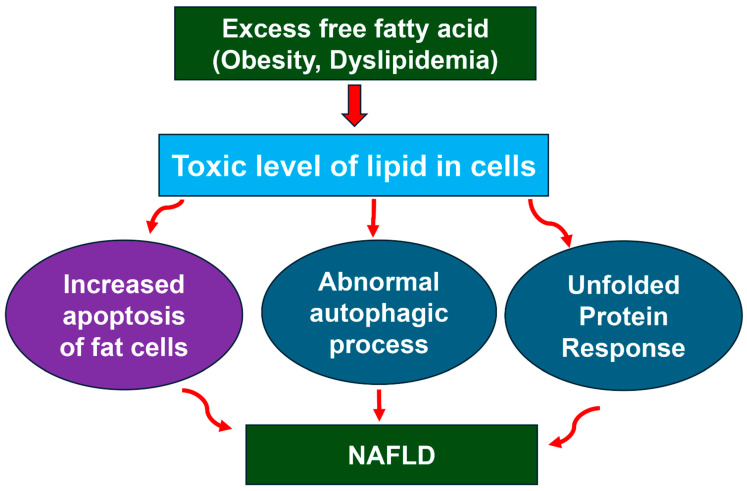
Molecular events leading to NAFLD. Excessive fat intake leads to obesity and dyslipidemia and overproduction of free fatty acids (FFA). Abnormal level of FFA causes lipo-toxicity, which can cause unfolded protein response, abnormal autophagy, and increased apoptosis of adipocytes.

In addition, autophagy also has a role in the mediation of inflammation by interacting with inflammatory signaling pathways, including the packaging and clearance of endogenous inflammasome activators and the modulation of cytokines and immune mediators [70,71]. For example, IL-1 and IL-17 production by macrophages infected with mycobacterium in Atg5^−/−^ mice increased, resulting in a pro-inflammatory state, according to the findings [72]. Atg5^−/−^ macrophages died at a higher rate than wild-type macrophages when exposed to oxidative and ER stress in the context of atherosclerosis, relative to the wild type [72]. All of these provide evidence of the role of autophagy in the mediation of the development and progression of NAFLD (Figure 4).

### 7.3. Increased Stress in Endoplasmic Reticulum

Overstimulation of the endoplasmic reticulum (ER) leads to an increased requirement for protein production to process excess fat and package it with lipoproteins for transport through body systems [73]. When the requirement for protein folding surpasses the ER’s capacity to handle the unfolded protein response (UPR), due to an accumulation of misfolded proteins in the ER complex, then stress occurs. Binding immunoglobulin protein (BiP), a molecular chaperone, plays a key role in assisting proper protein folding [73]. Under normal physiological conditions, BiP binds to and inhibits the activation of ER stress sensors. However, when unfolded protein levels rise, BiP dissociates from these stress sensors to focus on aiding protein folding. This dissociation activates three major UPR pathways: PERK (double-stranded RNA-dependent protein kinase/ER kinase), IRE1 (inositol-requiring enzyme 1), and ATF6 (activating transcription factor 6). Persistent activation of these pathways can result in a prolonged unfolded protein response [73]. UPR causes a maladaptive state of ER stress. In reality, saturated lipid compounds, such as palmitate, are harmful and have been found to impair the intactness of the ER membrane by saturating the phosphatidyl choline and phospholipids [73,74,75]. During ER stress, the transcription factor NF-B is activated through the PERK and IRE pathways, which overcomes the constitutively produced I (NF-B inhibitor). This allows NF-B to translocate more freely to the nucleus, so causing the formation of the proinflammatory genes to be activated. Finally, it has been demonstrated that ER stress in hepatocytes causes the activation of inflammasomes, which are proinflammatory multiprotein bio-complexes of the intrinsic immune network that triggers the response of the caspase-1 cascade to pathogenic microorganisms or host proteins, as shown in ob/ob mice. Lebeaupin et al. [76] demonstrated the activation of the LPS-induced inflammasome in mice through the elevated mRNA expression levels of several inflammasome components (Figure 3).

### 7.4. The Role of Oxidative Stress

Whenever steatosis evolves to steatohepatitis, it is believed that oxidative stress is a dominant factor in the development of the disease. An overabundance of nutrient supplements are fragmented in hepatocytes, which puts a critical strain on the electron transport chain in the mitochondria, bringing about the creation of free radicals (ozone, hydroxyl radical, hydrogen peroxide, and nitric oxide).

Reactive nitrogen species result from the abnormal breakdown of proteins with a concomitant increment in cellular oxidative stress [77]. On the other hand, the increased demand for electron transport chain proteins contributes to ER stress and dysfunction [77]. The formation of disulfide bonds during protein folding generates free oxygen radicals, leading to a feedback loop where ER stress exacerbates oxidative stress [77]. Studies have shown that while the livers of obese individuals exhibit increased fatty acid oxidation, their levels of fatty acid absorption and esterification remain similar to those observed in lean individuals [78].

### 7.5. Role of Lipo-Oxygenase Enzymes

The lipoxygenase (LOX) route is a potential novel pathway that might unite the three molecular pathways associated with NAFLD that have been identified. Based on lipidomics, researchers assessed two animal strains of NAFLD: control mice fed on a high-fat diet (HFD) and ob/ob mice fed on HFD. The results of the study indicated that the enrichment of triacyl glycerol and 18:1 fatty acid is the most noticeable change when compared to the control mice fed on standard chow [79]. It is thought that LOX enzymes are responsible for the conversion of polyunsaturated fatty acids in the plasma membrane, primarily arachidonic acid and linoleic acid, to create oxidized proinflammatory intermediates in the body [80,81]. The LOX route is a potential novel pathway that might unite the three different molecular pathways associated with NAFLD (Figure 4).

### 7.6. Association of Genetics and NAFLD

A strong link has been established between insulin resistance and NAFLD. Genetic variants linked to the severity of NAFLD have been detected in genome-wide association studies. It was discovered that the missense codon Ile148Met, which is present in the Patatin-like phospholipase domain-containing protein 3 (PNPLA3), was the origin of the mutation. Adiponutrin is a plasma protein that slows the progression of NAFLD [82] and causes weak hepatic triglyceride hydrolysis; however, it has nothing to do with insulin resistance or symptoms that resemble insulin resistance [83]. The Glu434Lys variation of PNPLA3, located at single nucleotide polymorphism rs2294918, has been shown to affect the PNPLA3 phenotype by reducing the amounts of PNPLA3 mRNA and protein in the liver. Glu167Lys is a variation of Glu167 at SNP rs58542926, and the quantity of fat in the liver is affected by inhibitors of the transmembrane 6 superfamily member 2 protein [84]; the carriers of this variation had a liver fat concentration that was 34% greater than the other variants [85]. Genetic variations in different ethnicities can lead to a higher severity of a disease, which can further lead to other complications as well (Figure 3).

## 8. NAFLD’S Association with Type 2 Diabetes Mellitus and Obesity

According to Tilg et al. [86], both hepatic and peripheral insulin resistance (IR) are closely linked to all types of NAFLD, and it gets worse as the illness advances. IR involves a decrease in glucose absorption due to the absence of an insulin-mediating pathway. The quantity of glucose generated rises as a result of increased gluconeogenesis and decreased hepatic glycogen synthesis. Insulin-stimulated hepatic glycogen production is triggered as a result. When this happens, a certain amount of fat in the liver shrinks. In obese people with type 2 diabetes (T2DM), NAFLD is linked to more severe dyslipidaemia, hyperinsulinemia, and insulin resistance in hepatic and adipose tissues than in non-obese people without NAFLD. Several theories have been proposed to explain the link between NAFLD and insulin resistance: Insulin resistance and hyperinsulinemia can promote hepatic fat build-up, or they can be generated directly by an excess of lipid availability, which subsequently leads to insulin resistance [86].

Being overweight is the most major risk factor for NAFLD, as evaluated by BMI and waist circumference, which is not only associated with the presence of NAFLD but also with its progression. Patients with NAFLD almost always have insulin resistance in their livers, which increases their risk of heart disease and increases their chances of getting T2DM in the future [86,87].

Insulin resistance (IR) in the liver and peripheral tissues is strongly linked to all forms of NAFLD, with severity increasing as the condition progresses. This resistance reduces glucose uptake by hepatocytes due to impaired insulin signaling, leading to elevated glucose production through increased gluconeogenesis and decreased hepatic glycogen synthesis. As a result, insulin-stimulated hepatic glycogen synthesis is activated, leaving less fat stored in the liver. Compared to non-obese individuals without NAFLD, obese individuals with type 2 diabetes (T2DM) and NAFLD experience more severe hyperinsulinemia, dyslipidemia, and IR in both the liver and adipose tissue [86]. The connection between NAFLD and IR may be influenced by multiple factors: liver fat accumulation might result from IR and hyperinsulinemia or may directly stem from an excess of available lipids, which subsequently induces IR [86].

Obesity, indicated by BMI and waist circumference, is the most critical risk factor for NAFLD. These measurements not only strongly correlate with the presence of NAFLD but also with its progression. Almost all NAFLD patients exhibit liver IR, significantly elevating the risk of heart disease and increasing the future likelihood of developing T2DM. However, IR can occur throughout the body, not just in the liver [86]. It is worth noting that IR is a precursor of T2DM [25] (Figure 3).

### 8.1. Genetic Association of NAFLD and T2DM

Despite the strong association between NAFLD and IR, genome-wide association studies have identified genetic variations linked to the severity of NAFLD that are not associated with insulin resistance or diabetes as explained above [66].

Inhibitors of the transmembrane 6 superfamily member 2 protein (TM6SF2) influence liver fat content, with individuals carrying this variation showing a 34% higher concentration of liver fat compared to other variants. However, these individuals exhibit improved insulin sensitivity in the absence of the variation. A mutation was identified in the gene encoding TM6SF2. Similarly, a genetic mutation in the ABHD5 gene, also known as CGI-58, reduces ABHD5 protein levels, leading to Chanarin-Dorfman syndrome and severe steatosis while preserving normal insulin sensitivity. ABHD5, an O-acyltransferase within the 1-acylglycerol-3-phosphate enzyme family, plays a role in fatty acid oxidation. When diacylglycerol accumulates in lipid droplets, it cannot facilitate PKC translocation, which is essential for the diacylglycerol-mediated inhibition of insulin signaling. This suggests a stronger dissociation between steatosis and IR in conditions like familial hypobetalipoproteinemia, where a genetic defect impairs hepatic triglyceride export. Consequently, intrahepatic triglyceride levels may serve as a marker of IR rather than its direct cause [86] (Figure 2).

### 8.2. Inflammation Pathway in the Development of Insulin Resistance

Increased understanding of the role of inflammatory pathways in the development of IR has led researchers to identify several new pathways contributing to this condition. However, the etiology of IR is complex and involves multiple mechanisms beyond inflammation. Aside from adipose tissue, the gastrointestinal tract with its significantly altered microbiota may be one of the early contributors to the development of IR and NAFLD, as well as affecting adipose tissue [88].

Insulin resistance (IR) is relatively new to scientific study. Hepatic IR in humans is believed to involve inflammatory and NF-κB pathways. However, NF-κB activation is relatively minimal in muscle, liver, and adipose tissue, where this gene is abundantly expressed. The IKK–NF-κB pathway plays a significant role in this process [89].

Insulin functions similarly in all cells by binding to a specific receptor and initiating a series of intracellular signaling events. Upon binding to insulin, the insulin receptor phosphorylates itself and various other proteins within the insulin receptor superfamily. IRS1 and IRS2 are crucial mediators of insulin signaling in the liver, helping regulate insulin sensitivity. The disruption of insulin signaling, likely due to inflammatory mediators, is a key pathological process behind IR. Elevated levels of pro-inflammatory cytokines and transcription factors are found in various organs, including adipose tissue and the liver, in individuals with obesity and related conditions [90].

Individuals with IR engage specific pro-inflammatory pathways that produce cytokines such as tumor necrosis factor-alpha (TNF-alpha) and interleukin-6 (IL-6). It is believed that the nuclear factor-kappa B (NF-κB) and downstream inflammatory signaling pathways play a significant role in IR. The IKK (I-κB kinase) complex is essential in this process, as it can activate NF-κB by phosphorylating the inhibitory molecule I-κB, thus blocking its inhibitory action. The enzymes involved include IKK-alpha, IKK-beta, IKK-gamma, and TANK-binding kinase 1 (TBK1) [91].

In 2001, Yuan and colleagues identified the IKK pathway as a potential target for addressing IR [89]. Two subsequent studies linked IKK expression in the liver to IR. Researchers created a transgenic mouse model of chronic hepatic inflammation by constitutively expressing IKK specifically in hepatocytes, leading to low-level activation of NF-κB. These mice developed a condition similar to T2DM, alongside mild systemic IR. Arkan et al. found that animals lacking IKK in either hepatocytes or myeloid cells had similar results. Although mice with a liver-specific IKK deletion maintained insulin responsiveness in the liver after a high-fat diet or when crossed with ob/ob mice. They developed IR in muscle and adipose tissue after a high-fat diet. Interestingly, mice lacking myeloid IKK-beta also preserved systemic insulin sensitivity. Thus, hepatocyte-specific overexpression or activation of NF-κB is associated with IR and can mimic characteristics of fatty liver disease, highlighting the significant role of inflammation in IR [92].

Insulin resistance was shown to be connected with elevated blood sugar levels; however, the increase in fatty acid oxidation did not occur simultaneously with the rise in mitochondrial respiratory chain (MRC) activity, as previously thought. The long-chain acylcarnitine/carnitine ratio was found to be higher in patients with NASH when compared to healthy controls, whereas MRC complexes I through IV were found to be lower, suggesting that the mitochondrial respiratory chain is unable to keep up with the rate of fat oxidation, thereby increasing oxidative stress in hepatocytes [93]. Patients with NASH had higher levels of lipid peroxidation products, such as malondialdehyde (MDA), hydroxynonenol (HNE), oxidized LDL (ox-LDL), and thiobarbituric acid-reacting substances (TBARS), in their plasma compared to patients with steatosis alone, suggesting that oxidative stress may have played a role in the progression from steatosis to NASH [94].

Furthermore, when comparing individuals with NASH to those with steatosis alone, the levels of HNE and 8-hydroxy deoxyguanosine staining are much greater in the liver tissue of the NASH patients [95]. According to the findings, the intensity of 3-nitrotyrosine staining in the livers of people with NASH was higher than in those of subjects with steatosis alone, and it was higher than that of subjects with steatosis and NASH combined. Studies have also delved into the possibility of employing serum thioredoxin as a non-invasive diagnostic tool. As a marker of NASH, it was found to be considerably higher in individuals with NASH compared to those with steatosis or diabetes [96].

## 9. Future Options and Treatment of NAFLD

Improving one’s lifestyle is an efficient tool to prevent and cure NAFLD. Maintaining a healthy body weight, the first step in addressing NAFLD, requires a well-balanced diet and frequent physical activity [97,98]. The Mediterranean diet is low in calories and can aid in weight loss, cholesterol build-up in the liver, and IR. It can also raise monounsaturated and n-3 polyunsaturated fat levels in the blood while lowering saturated fat levels [97,99]. Weight loss surgery, often known as “bariatric surgery”, is a kind of surgery that helps people lose their fat and weight, which is regarded as the most effective treatment for obesity and diabetes because it reduces food absorption while also modifying gut hormone release and metabolic dysfunction in patients.

A person with type 2 diabetes who used SGLT2 inhibitors, GLP-1 receptor antagonists, and insulin exhibited a lower frequency of NAFLD than the general population [97,100].

It is being evaluated as a therapy option for NAFLD/NASH that anti-diabetic or anti-obesity drugs, such as pioglitazone and saroglitazar, are reprogrammed. The development of fibrosis that occurs in NAFLD and other liver diseases can be slowed down by anti-fibrotic drugs. Polyphenols, which act as anti-inflammatory and antioxidant agents, have been shown to have a protective impact on the treatment of liver disease, and the intake of polyphenol-rich diets has been shown to have favorable benefits in the treatment of NAFLD patients. Increasing the consumption of lignans, a wide category of low molecular weight polyphenols found in plants, such as whole grains, can lower the risk of developing NAFLD [2,97].

## 10. Conclusions

Ethnic and cultural differences can have a profound impact on the progression and severity of NAFLD and T2DM, either mitigating or exacerbating their effects. Factors such as diet, physical activity, religious practices, and various molecular, genetic, and clinical characteristics all contribute to these variations. The rapid rise in non-alcoholic fatty liver disease (NAFLD) in the United States and worldwide is fueling an increase in severe comorbidities, including cardiovascular diseases, diabetes mellitus, and metabolic syndrome. This growing health crisis serves as a critical warning for both patients and healthcare providers. Physicians must account for the unique cultural factors associated with different ethnic groups when managing patient care. Tailoring treatment approaches accordingly can lead to improved health outcomes and contribute to a healthier global population.

## Figures and Tables

**Table 1:** Worldwide prevalence of NAFLD.

#	Region/Country	Prevalence (%)	Reference
1	Worldwide	25.2	[2,8]
2	Middle East and North Africa	40.0	[9,10]
2	USA	34.0	[8,11,12,17]
3	Asia	29.0	[14]
4	South America	59.0	[8,9,10,13]
5	Africa	13.5−25.2	[8,10]
6	United Kingdom	20.7	[16]

It is noticeable in the table that NAFLD is most common in the USA, South America, Africa, and the MENA region. MENA = Middle East and North Africa.

## Data Availability

All data used have been made available in the manuscript. The study did not report any dataset.
